# Exercise based reduction of falls in communitydwelling older adults: a network meta-analysis

**DOI:** 10.1186/s11556-023-00311-w

**Published:** 2023-01-28

**Authors:** Tim Wiedenmann, Steffen Held, Ludwig Rappelt, Martin Grauduszus, Sofie Spickermann, Lars Donath

**Affiliations:** Department of Intervention Research in Exercise Training, Institute of Exercise Training and Sport Informatics, Am Sportpark Müngersdorf 6, 50933 Cologne, German Sport University Cologne, Cologne, Germany

**Keywords:** Elderly, Training, Seniors, Balance, Strength, Fall prevention

## Abstract

**Background:**

Traditional meta-analyses with pairwise direct comparison revealed that a variety of exercise-based training interventions can prevent falls in community-dwelling older adults. This network meta-analysis adds value by comparing and ranking different exercise training strategies based on their effects on fall risk reductions determined by analysis of direct and indirect comparisons.

**Methods:**

The studies included in this network meta-analysis were identified through a comprehensive search in five biomedical databases (PubMed, SportDiscus, CINAHL, Web of Science and EMBASE). We included (randomized) controlled trials (RCTs) that compared the occurance of fall events in older adults who received different interventional treatments.

**Results:**

Seventy six comparisons from 66 RCTs with 47.420 (61% male / 39% female) participants aged 77 ± 4 (68 – 88) years were included in this network meta-analysis. The network model revealed low heterogeneity (I^2^ = 28.0, 95%CI 1.0 to 47.7%) and inconsistency (Q between designs = 15.1, *p* = 0.37). Postural control training was found to be most effective in preventing falls (Postural Control Training: (home): Risk Ratio (RR) = 0.66, 95%-CI [0.49; 0.88], P-score = 0.97;Postural Control Training: RR = 0.82, 95%-CI [0.75; 0.91], P-score = 0.82). Combined and multifactorial interventions also display a robust but smaller effect (RR = 0.88–0.93, P-score = 0.65–0.47).

**Conclusion:**

Physical activity that includes balance training presents itself to be the most effective. Multifactorial approaches are well investigated but could be slightly less effective than isolated postural control training.

**Supplementary Information:**

The online version contains supplementary material available at 10.1186/s11556-023-00311-w.

## Background

Falls are a serious health concern and a major cause of morbidity and mortality in community-dwelling older adults [1]. Approximately one in three older adults above the age of 65 fall at least once a year and half of them are recurrent fallers [2, 3]. Considering the growing life expectancy in western societies and the increasing risk of falls during the later years in life, resulting health care costs caused by injurious falls will continue to increase until the end of the current century [4]. The prevention of falls is therefore not only of utmost importance for maintaining independency of daily living, maintaining wellbeing and quality of life in older adults [5] but also an urgent economic challenge for the healthcare system.

Available clinical practice guidelines for fall prevention in the older population underpin the importance of physical activity and exercise [6]. Observational, interventional and meta-analytical studies report beneficial effects of physical activity in general and specific exercise training in particular for the prevention of falls [7–11]: An impactful meta-analysis by Sherrington and colleagues, for example, found a reduction of falls by about 23% and in number of fallers by around 15% following physical physical training [8].

The majority of the included exercise-based fall prevention studies focus on balance exercises or resistance training. While tendencies favouring balance exercises for the prevention of falls can be observed [8], it is not entirely clear whether balance exercise alone or in a combination with multiple exercise forms is most effective for reducing fall risk. A network meta-analysis enables the calculation and comparison of treatment estimates from direct and indirect evidence by using a common comparator that multiple interventions compare against. This allows for the estimation of comparative effects for a large variety of interventions, including some comparisons that have never been made directly [12, 13]. Hence, this network meta-analysis provides comprehensive effect rankings that can help to find the physical training program that has the strongest effect on reducing the number of falls.

Against this background, the aims of this network meta-analysis are: (i) to rank different physical activities based on their effect on fall prevention in older adults and (ii) to analyse which form of exercise is most suitable for fall prevention. The outcome of this network meta-analysis can help to find a training program that could prevent health care costs from rising and increases quality of life in the later years for older adults.

## Methods

### Search and screening procedures

This network meta-analytical review was registered [14] and conducted in accordance with the Preferred Reporting Items for Systematic Reviews and Meta-Analyses for Network Meta-Analyses (PRISMA-NMA) [15]. The literature search and screening processes were independently conducted by two researchers (SS and MG). Five health-related, biomedical and psychological data-bases (PubMed/MEDLINE; SPORTDiscus EBSCO, the Cumulative Index to Nursing and Allied Health Literature (CINAHL EBSCO); Web of Science and EMBASE) were screened from inception of the respective journals until December 3rd 2021. Relevant search terms (operators) were combined with Boolean conjunctions (OR/AND) and applied on three search levels (Table 1). In addition, tracking of cited articles and hand searching of relevant primary articles and reviews were also carried out. Duplicates were removed and the remaining studies underwent a manual screening. The remaining studies were gradually screened using (1) the titles, (2) abstracts and (3) full-texts of the potentially eligible articles. The following inclusion criteria based on the PICOS approach [population (P), intervention (I), comparators (C), main outcome (O), and study design (S)] were applied: Full-text article published in English in a peer-reviewed journal; Participants were community-dwelling, independently living people involved in studies with a mean age of at least 65 years and an age larger than 60 years when subtracting one standard deviation from the studies mean age, without additional diseases (e.g., stroke, chronic stroke, Parkinson’s disease, multiple sclerosis, dementia, hip fractures or other fractures) or an acute or chronic mental or physical illness (such as cancer, depression, mild cognitive impairment, diabetes mellitus or COPD) (P). All studies that included at least one exercise intervention group and one control or another exercise intervention group were eligible. To rule out crosstalk effects, supplement and medication studies were excluded (I). Comparators were groups with no or light physical exercise (C). Documentation of the incidence of falls, to estimate the risk ratio (RR), for at least six months, regardless of whether they were documented within the intervention period, as a follow-up after the intervention, or during the intervention and in a follow-up period. A fall was defined as a subject’s unintentionally coming to rest on the ground or at some other lower level, not as a result of a major intrinsic event (e.g. stroke or syncope) or overwhelming hazard [16] (O). Furthermore, the studies had to be two- or multiarmed randomized controlled trails (S). The exclusion criteria were: (1) No adequate control conditions, which made integration into the network impossible. (2) The use of an alternative supporting structures or systems such as an exoskeleton.

**Table 1:** Search strategy

Search level	Search terms with Boolean operators
Search #1	“falls” OR “faller*”
Search #2	#1 AND (“aged” OR “senior*” OR “elder*” OR “old” OR “aging” OR “ageing” OR “postmenopausal” OR “community-dwelling”)
Search #3	#2 AND (“randomized” OR “placebo” OR “trial”)

### Assessment of methodological quality of the studies

The methodological quality (including risk of bias) of the included studies was independently rated by two researchers (SS and MG) using the PEDro (Physiotherapy Evidence Database) scale [17]. The PEDro scale consists of 11 dichotomous (yes or no) items, in which the criteria 2–9 rate randomization and internal validity and the criteria 10–11 rate the presence of statistical replicable results. Criterion 1 relates to the external validity and is not being considered in the PEDro score sum. A PEDro score ≥ 6 from 0 to 10 [17] represents a high quality study.

### Data extraction

Relevant data (required for calculating effect sizes) were extracted independently by two researchers (SS and MG) using a standardized extraction spreadsheet (Microsoft Excel) adapted from the Cochrane Collaboration [18]. To estimate the effect of exercise on the incidence of fallers, the number of fallers and non-fallers in each intervention group were extracted. If these values were not available, authors were contacted and asked to provide the data. In addition to the outcomes, relevant information about the included studies (author, year of publication, number of participants) and their interventional design (weeks, frequency, duration per session, type of intervention and control condition) were also recorded. For the simplification of the network, similar treatments haven been summarized in (i) Active Control; (ii) Combined Postural Control Training;; (iii) Endurance Training; (iv) Inactive Control; (v) Multifactorial Training; (vi) Postural Control Training; and (vii) Resistance Training. Thereby, ‘Postural Control Training’ was defined as balance, coordination and/or multitask training. ‘Combined Postural Control Training’ was chosen if resistance or endurance training were performed additionally to postural control training as it is the case in the popular OTAGO exercise program, for example. ‘Multifactorial Training’ was categorized as forms of training that included other non exercise related factors influencing the risk of falls (such as home hazard management and visual, educational or behavioral interventions) in addition to postural control training. For an additional differentiation within the three treatment summaries (Postural Control Training, Combined Postural Control Training and Multifactorial Training) the label “home” indicated whether the corresponding intervention was conducted as home-based training. The ‘active control’ treatment features interventions that are not thought to influence the outcome of falls such as light stretching and relaxation.

### Statistical analysis

The RR were calculated for all interventional treatments by dividing the incidence of the intervention group by the incidence of the reference group. If values for the (RR) were already given, these data were used. Additionally mean error and 95% confidence interval (95%-CI) were evaluated. Subsequently, a network model was computed [19, 20]. Therefore a frequentist approach was chosen. In order to visualize the networks, a network graph was created. The estimations of treatment effects were calculated based on a random effects model [21]. Thereby, the Inactive Control served as the reference treatment. A ranking was created based on the P-score of the individual treatments. The P-score represents the means of one sided *p*-values under normality assumption in a frequentist NMA [19]. It is interpreted as the mean extent of certainty that one intervention is superior to any other and is analogous to the surface under the cumulative ranking curve (SUCRA) [22] values of Bayesian NMA [19]. P-scores range from 0 to 100% with 0 and 1 being the theoretically worst and best treatment, respectively. Additionally, a forest plot was created to further visualize the ranking and effects of the treatments. The decomposed Q-statistics (within and between designs) were used to interpret potential heterogeneity and inconsistency. Heterogeneity was further quantified by I^2^ [23]. Funnel plots were created to check potential publication bias. All calculations and presentational figures were made using the R software (version 4.1.1; The R Foundation for Statistical Computing) and the package ‘netmeta’ [20]. Values in the written text are presented as *mean (SD)* if not stated otherwise.

## Results

### Study characteristics and quality

**Fig. 1:**
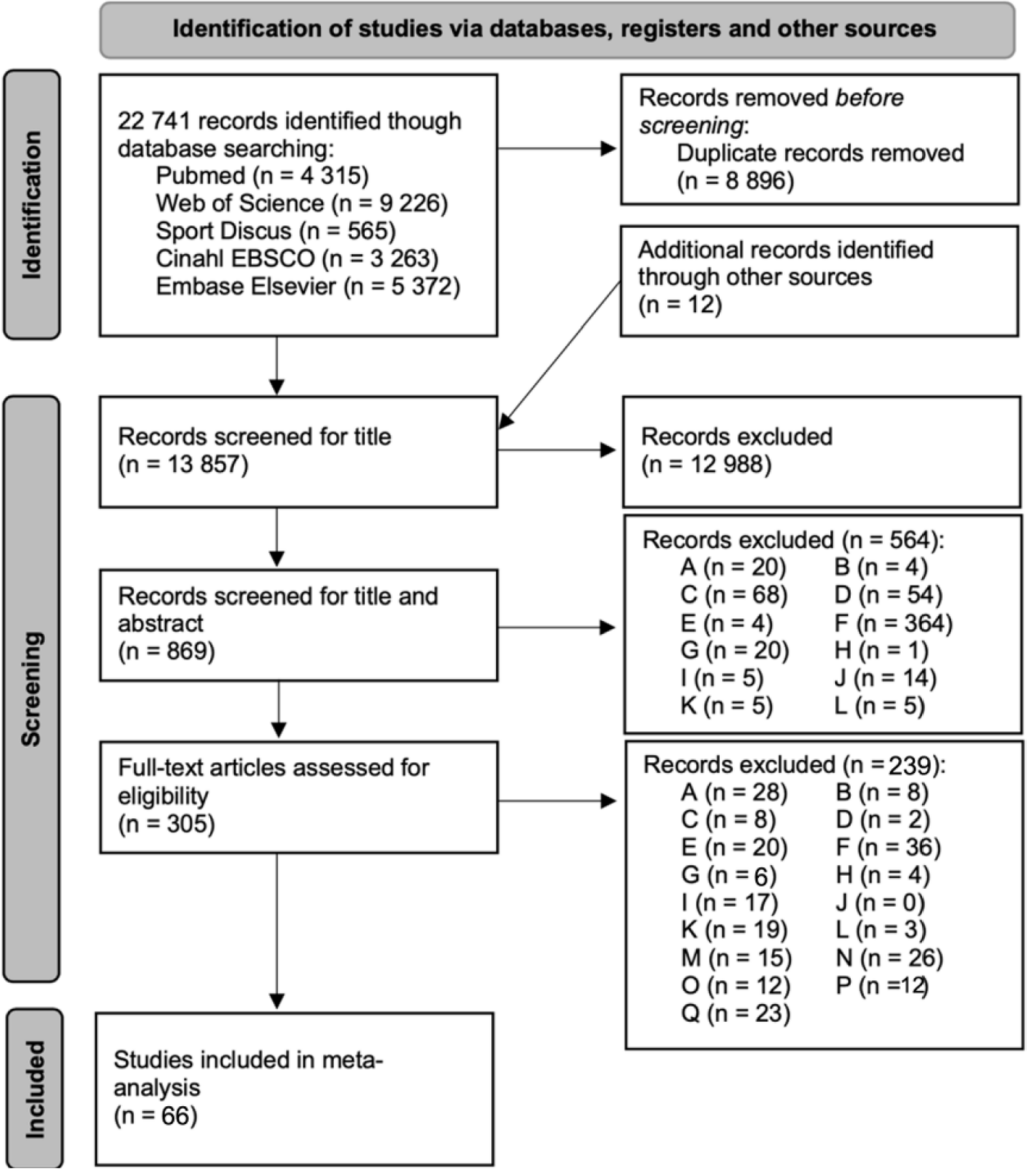
Flow chart of the different phases of study screening and selection. **A**: no RCT; **B**: wrong age; **C**: study without community dwelling people; **D**: population with high risk of falling (stroke, Parkinson’s disease, multiple sclerosis, dementia, hip fracture, severe visual impairment, mild cognitive impairment, fractures); **E**: Study in which Exercise is not measured in a controlled way; **F**: no incidence of fallers; **G**: study without exercise; **H**: Study with medications or supplements; **I**: duplicates; **J**: Chronic or acute illnesses, e.g. depression, diabetes mellitus, COPD, cancer, mental & physical disabilities; **K**: Measurement of rate of fallers under 6 months reported; **L**: wrong language; **M**: no full text; **N**: Study results reported by another study; **O**: only abstract, poster etc.; **P**: Intervention groups in network not comparable; **Q**: not responded after 3 reminders for data request

**Fig. 2:**
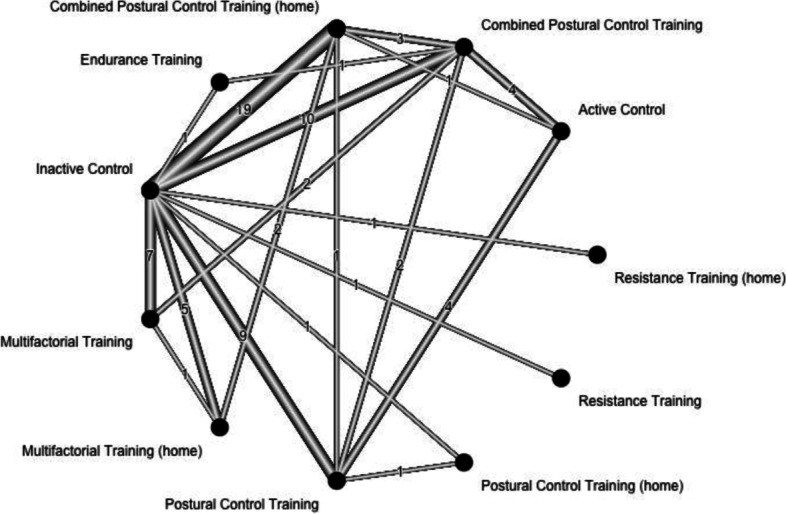
Network plot with all of the direct comparisons represented by the linking lines. The number of comparisons is represented by the number on the lines. The thickness of the lines increases with the number of comparisons

Overview of screening and study selection are presented in Fig. 1. Details to all selected studies are given in Supplementary Table 1. Included trials (47.420 participants; 61 % male; 39 % female) enrolled on average 624 ± 1426 participants per study (range 21 to 6580) with an average age of 77 ± 4 years (range from 68 to 88 years of age). The average study quality was high as indicated by a PEDro score of 7.1 ± 1.0 (range 5 to 9). Apart from 5 three armed designs [24–28], all remaining studies employed a two-armed design [29–88]. Data from 66 studies representing 76 pairwise comparisons were included. The most common comparison was ‘Inactive Control’ vs. ‘Combined Postural Control Training (home)’ (*n* = 19), followed by ‘Inactive Control’ vs. ‘Combined Postural Control Training’ (*n* = 10), and ‘Inactive Control’ vs. ‘Postural Training’ (*n* = 9). All of the pairwise comparisons are depicted in the network plot in Fig. 2. The network model revealed low heterogeneity (I^2^ = 28.0, 95%CI 1.0 to 47.7%) and non-significant inconsistency (Q between designs = 15.1, *p* = 0.37).

### Risk of bias

**Fig. 3:**
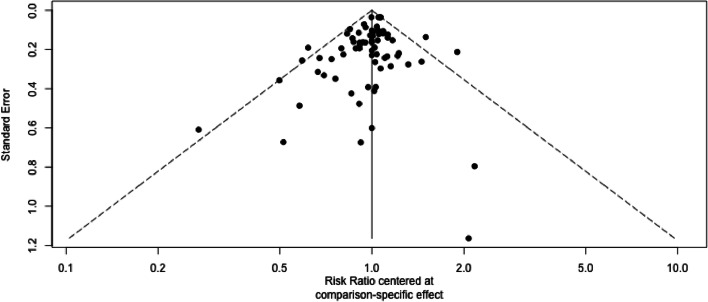
Funnel plot

The evaluation of the funnel plot revealed no considerable asymmetries that would indicate a potential risk of bias. (Fig. 3). Only four studies are located outside of the inverted funnel [33, 61, 78, 80]. Three of them investigated ‘Combined Postural Control Training (home)’ [33, 78, 80] and one investigated ‘Multifactorial Training (home)’ [61] all of them had an ‘Inactive Control’ Group as Comparator.

### Treatment-ranking

**Fig. 4:**
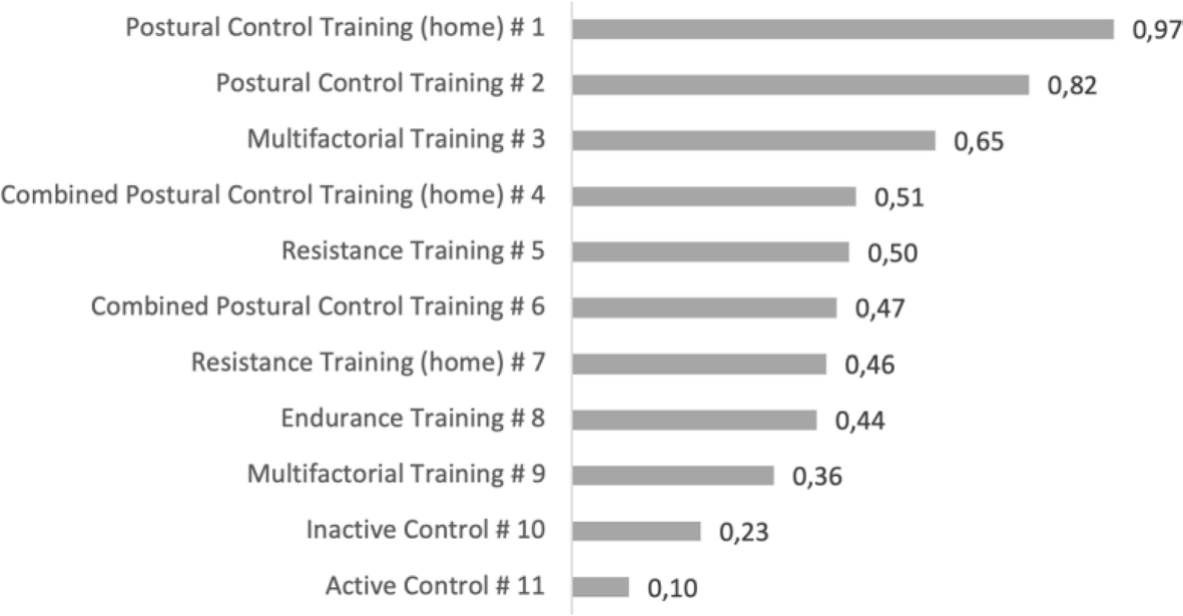
P-score ranking

**Fig. 5:**
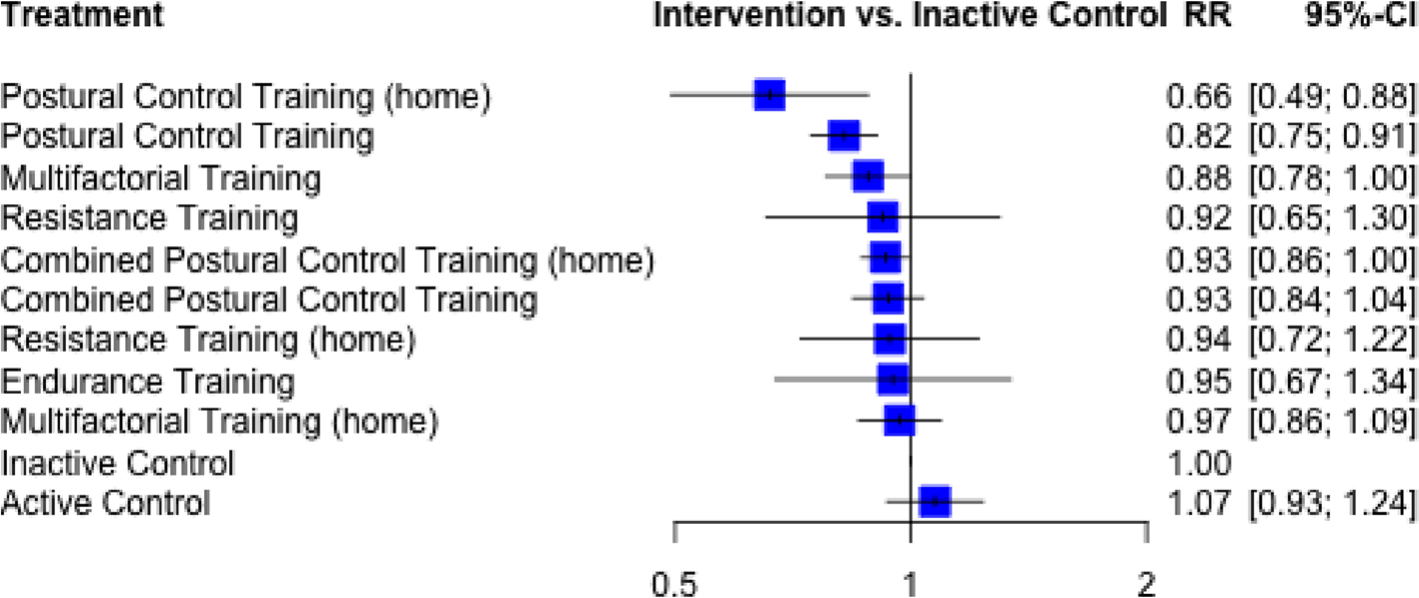
Forest plot. RR: risk ratio; 95%-CI: 95% confidence interval

The ranking of the different treatments is depicted in Fig. 4. The RR that the P-score rankings are based upon and their 95%-CI are depicted in Fig. 5. ‘Postural Control Training (home)’, and the regular ‘Postural Control Training’ rank the highest with respective P-scores of 0.97 and 0.82. The two highest ranked exercise modes display low RR from 0.60 to 0.82 for ‘Postural Control Training (home)’ and regular ‘Postural Control Training’ respectively. The third to eighth ranked interventions are ‘Multifactorial Training’, ‘Combined Postural Control Training (home)‘, ‘Resistance Training’, ‘Combined Postural Control Training’, ‘Resistance Training (home)’ and ‘Endurance Training’ with similar P-scores ranging from 0.65 to 0.44 and RR from 0.88 to 0.95. ‘Multifactorial Training (home) is third to last with a P-score of 0.36 and a RR of 0.97. ‘Inactive Contol’ and ‘Active Control’ rank the lowest among the treatments with respective P-scores of 0.23 and 0.10.

## Discussion

This network meta-analysis was conducted to extend meta-analytical knowledge by analysing the direct and indirect comparison of different forms of physical training and its effects on the relative fall risk in older adults. Compared to a traditional meta-analytical approach with pairwise direct comparison, our network meta-analysis provides additional evidence by ranking the effects of numerous different training interventions that were not comparable in previous analyses. Our key finding was that balance and strength focused exercise modes are the most beneficial for the prevention of fall events.

Among all included exercise modes, the balance type exercise modes revealed the lowest relative risk for a fall event and was therefore placed highest in the P-score ranking outranking the resistance training and resistance training combined approaches. These findings are aligned with previous findings and reflect the majority of available clinical guidelines but go beyond them [6, 8]. In the most recent and impactful meta-analysis, Sherrington and colleagues concluded that physical training, if challenging enough, leads to a reduction of falls of approximately 23% [8]. They further emphasized that training involving balance exercises is most beneficial for the prevention of falls. This is well in line with the treatment ranking of the postural control training in our network meta-analysis and the 18 - 34% reduction in fall risk implied by the computed risk ratio of our analysis. Additionally, our current network meta-analysis displays that these reductions in fall risk are also present when postural control training is performed at home and largely unsupervised.

‘Multifactorial Training’, ‘Combined Postural Training (home)’ and ‘Combined Postural Training’ are associated with a slight reduction of fall risk and a high precision of data, indicated by narrow confidence limits. These findings suggest that an interventional approach with multiple different exercise modes or other non-exercise related factors are inferior in magnitude but robust in effect occurance of fall risk reductions compared with the most beneficial postural control training interventions when they are performed isolated. This is at least partially in line with a meta-regression and meta-analysis from 2007. Campbell et al. (2007) found that single factorial interventions had similar but slightly favourable effects compared to multifactorial interventions [89] when it comes to the prevention of falls. However, it might be reasonable to assume that multifactorial interventions result in broader adaptations than single factorial approaches. As a result, larger effects could potentially be stimulated when the interventions are performed with an adequate intensity, frequency and duration. Thus, although the effects of the combined interventions are inferior to the best single factorial interventions, the high precision of the data combined with the large amount of included evidence (51 direct comparisons) leads to the assumption that some combined and multifactorial interventions might be a valuable alternative suited for a large variety of populations with different demands. The value of multifactorial interventions might even be higher when the interventions are designed with different domains that are specific to the needs of the individual patient. A very recent statement with new guidelines discussed by experts from different fields who were led by Montero-Odasso and colleagues emphasizes the efficacy of multidomain interventions and the assessment of individual needs [90]. It is possible that with a new and more personalized approach to a multifactorial training the effects of the intervention would be larger. In contrast, ‘Resistance Training’, Resistance Training (home), ‘Endurance Training’, and ‘Multifactorial Training (home)’, “and do not have a robust positive effect on the relative risk of falls. While these interventions display a risk ratio that is also slightly lower than the control, they are accompanied by verylarge confidence limits. There is a noticeable difference in the risk reduction between ‘Multifactorial Training’ which places third in the treatment ranking and its home base counterpart ‘Multifactorial Training (home)’ which has no clear positive effect on the reduction of fall risk. A similar difference but considerably smaller in magnitude is observed for ‘Resistance Training’ and ‘Resistance Training (home)’. These observations are in line with other meta-analyses which found that home based exercise interventions do not have a clear benefit for the prevention of falls [91] and are less effective than supervised programs [92]. A possible explanation for these shared findings might be that the compliance, adherence and the effort exerted in training are not sufficient when an intervention is performed largely or completely unsupervised at home. The two interventions that fall out of line are the postural control training and the combined postural control training which combines strength or endurance training with the former. For these two approaches the unsupervised home training displays a larger effect than the supervised intervention or the same effect for postural control training and the combined intervention respectively. However, there are only two studies [38, 88] directly comparing ‘Postural Control Training (home)’ with other interventions included in this analysis and therefore the findings should be interpreted with caution. It is reasonable to assume that with increasing evidence the effect of ‘Postural Control Training (home)’ would regress in the direction of its fully supervised counterpart that ist not practiced at home.

### Strengths & Limitations

Some limitations need to be addressed. One limitation was the heterogeneity in duration of the studies and follow up periods. Future research should investigate how the length of an intervention period influences the effects of the different training modes. Another limitation was the amount of evidence that is available for the analysis. For some of the investigated interventions data precision is very low. This is likely due to the sparsity of studies and overall evidence that was included in the analysis for certain interventions. When data precision is as low as it is for the resistance and endurance training interventions one has to be cautious when interpreting the results. Emerging studies should, however, help to solve this issue. Other than the sparsity of data for some of the interventions, the quality of the included evidence is good. This is indicated by the overall high PEDro scores of the included studies and the funnel plots which did not show any signs of bias. Together with the high quality of evidence, the very large amount of analysed evidence is a definite strength of this analysis.

## Conclusion

The evidence summarized in this network meta-analysis shows that balance training is the mode of physical activity or exercise that has the strongest positive impact on fall risk. For interventions that combine different modes of exercise or other non-exercise interventions the influence becomes less strong (up to about 12% reduction of fall risk) but still rather clear due to narrow confidence limits. With the exception of balance training type exercise and the combined postural training approach, training performed at home was not as effective as training that was completely supervised. Future studies should investigate the role of training intensity and effort as well as the effects of multimodal exercise training over longer study periods up to ≥ 1 year.

## Supplementary Information

**Additional file 1.** Supplementary table with all the studies and their information.

## Data Availability

The datasets used and/or analysed during the current study are available from the corresponding author on reasonable request.
